# Research progress on the adverse effects of high-altitude environment to the male reproductive system: a review study

**DOI:** 10.3389/fendo.2025.1573502

**Published:** 2025-05-14

**Authors:** Dong-Dong Meng, Yin-Dong Kang, De-Hui Chang

**Affiliations:** Department of Urology, The 940th Hospital of Joint Service Support Force of Chinese People's Liberation Army, Lanzhou, Gansu, China

**Keywords:** high-altitude environment, male reproductive system, reproductive hormones, testicular tissue, sperm quality

## Abstract

An increasing number of people are being exposed to high-altitude environments as they become more important in economic development, resource exploitation, and other areas. This review is focused on the impact of the high-altitude environment on the male reproductive system. In high-altitude areas, the unique conditions lead to complex and significant changes in male reproductive hormone levels. The secretion of GnRH is inhibited, which in turn affects the levels of FSH and LH, ultimately influencing testosterone synthesis and secretion, thus disrupting the normal endocrine regulatory network. Testicular tissue also shows marked morphological changes. The seminiferous tubule structure becomes disordered, and the number and function of spermatogenic and interstitial cells are damaged. These alterations have a direct impact on sperm quality, resulting in a decrease in sperm density and motility, an increase in the deformity rate, and damage to genetic material integrity. Additionally, sexual function is affected, with symptoms such as decreased libido and erectile dysfunction being common. The underlying mechanisms involve oxidative stress damage, an abnormal increase in apoptosis, and enhanced autophagy. Nevertheless, current research, especially human-based studies, is restricted by small sample sizes and insufficiently in-depth exploration of these mechanisms.

## 1 Introduction

In the progression of global economic and social development, high-altitude areas have assumed increasing importance in mineral exploitation (1), tourism expansion (2), military garrisoning (3), and numerous other fields due to their abundant natural resources and distinctive geographical strategic positions. In recent years, with mineral development projects in high-altitude regions continuously growing, more and more people are settling or frequently traveling in high-altitude areas. The environment in high-altitude regions is characterized by low atmospheric pressure (4), low oxygen partial pressure (5), low temperature (6), and intense ultraviolet radiation (7), posing formidable challenges to human physiological functions.

In high-altitude areas, the atmospheric pressure and the closely related oxygen partial pressure drop significantly with increasing altitude (8). Hypoxia is common in high-altitude regions like the Qinghai-Tibet Plateau and the Andes Mountains, having a profound impact on human physiological functions by affecting energy metabolism, reducing low-temperature tolerance, and interacting with strong ultraviolet radiation (9). There is a highly significant negative correlation between air temperature and altitude in high-altitude areas (10), and this low-temperature environment affects various aspects of the human body including basal metabolism and physiological functions. Also, due to the thin air, ultraviolet radiation attenuation is much lower than in low-altitude regions (11), resulting in a substantial increase in ultraviolet radiation intensity, and excessive ultraviolet irradiation can damage biomacromolecules and skin tissues (12).

The male reproductive system, being a crucial physiological system for maintaining human reproduction and individual quality of life, has attracted significant attention regarding its health status in high-altitude environments (13–15). Male reproductive health is not only directly related to an individual’s fertility but also plays an essential role in family stability and the optimization of the social population structure (16). Therefore, delving deeply into the potential effects and mechanisms of the high-altitude environment on the male reproductive system is of great significance for effectively safeguarding the reproductive health of men in high-altitude areas, scientifically formulating targeted protection strategies, and promoting the advancement of research in related fields.

## 2 Impacts of high-altitude environment on the male reproductive system

### 2.1 Impact on reproductive hormone levels

Long-term exposure to the high-altitude hypoxic environment induces intricate and clinically significant changes in male reproductive hormone levels (17). Gonadotropin-releasing hormone (GnRH), a pivotal hormone secreted by the hypothalamus, plays a crucial “directing” role in the reproductive endocrine regulatory network, and its secretion process is notably inhibited by the hypoxic environment (18, 19).

The neurons in the hypothalamus are highly sensitive to oxygen partial pressure. In a hypoxic state, the normal metabolism and signal transduction processes of neurons are severely disrupted, resulting in the suppression of GnRH synthesis and release. Previous studies have demonstrated that in a specific high-altitude environment, such as at an altitude of 4350 meters, the FSH level in 8 healthy male subjects decreased by 17% (19). In another study involving 40 healthy subjects (21 men) within the altitude range of 550 meters to 7050 meters, the results revealed that the male FSH level continuously declined from 5.8 ± 5.9 mU/L at 550 meters (baseline level) with increasing altitude, reaching 3.3 ± 2.1 mU/L at 7050 meters, with a reduction of up to 40%. Simultaneously, LH also decreased significantly from the baseline to 7050 meters, by approximately 50% (20).

The decline in GnRH secretion further affects the pituitary’s secretion of follicle-stimulating hormone (FSH) and luteinizing hormone (LH), and FSH and LH are essential for testicular spermatogenesis and testosterone synthesis and secretion (21, 22). FSH primarily acts on the Sertoli cells in the seminiferous tubules of the testis to promote their maintenance of normal function and provide necessary nutrition and support for the spermatogenic process (23–25). LH mainly acts on the Leydig cells of the testis to stimulate their synthesis and secretion of testosterone (26–29). Hence, when the levels of FSH and LH decrease, the functions of the Sertoli cells and Leydig cells in the seminiferous tubules of the testis are damaged to varying degrees, thereby influencing sperm production and testosterone secretion (22, 30, 31).

Testosterone, as the core hormone of male reproductive function, is also affected by multiple factors in its synthesis and secretion (32, 33). In von Wolff et al.’s study, testosterone concentration remained relatively stable at most altitudes, with minimal fluctuations. However, at 7050 m, testosterone concentration decreased significantly, paralleling the decline in LH concentration (20). Previous research by Benso et al. also reported a significant reduction in testosterone concentrations in eight mountaineers at the 5200-m base camp post-Mount Everest climb (34). Nevertheless, due to the small sample size and confounding effect of excessive exertion, the interpretability of those results was limited. In summary, as altitude rises, particularly at extreme altitudes, testosterone concentration is significantly affected and generally decreases. This change is likely associated with alterations in other hormone levels and altitude-related environmental factors (35).

### 2.2 Impact on testicular tissue morphology

Animal experiments have provided crucial evidence for elucidating the morphological changes of testicular tissue in a high-altitude environment. For example, in the experiment by Li et al., 3-week-old male Wistar rats were housed in a temperature- and humidity-controlled animal room, and a low-pressure hypoxic environment simulating an altitude of 5000 meters was created using a hypobaric hypoxia chamber (36, 37). The experimental outcomes showed that compared with the control rats in the normal environment, the seminiferous tubule structure of the rats in the low-pressure hypoxic group was significantly disordered, the arrangement of spermatogenic epithelial cells lost its normal order, and the number of spermatogenic cells at all levels decreased sharply, especially the number of spermatocytes and spermatids. Additionally, Li et al.’s study also confirmed that the testosterone levels in the low-pressure hypoxic group were significantly lower than those in the normal environment group (13).

Hypoxia is closely related to the testis and significantly affects testicular function and reproductive health (38, 39). In high-altitude environments, except for certain indigenous populations, human reproductive function is affected (40–42). Animal experiments show that hypoxia can change the morphology of testicular tissue and testosterone levels (43, 44). When the testis is hypoxic, HIF-1α accumulates (45, 46), triggering vascular changes, including angiogenesis. The effect of hypoxia on testicular steroidogenesis is complex, with testosterone levels rising first (47) and then falling (34). Chronic intermittent hypobaric hypoxia damages testicular tissue, reduces sperm quality, and decreases testosterone levels (42). Hypoxia also increases testicular temperature, generates ROS (48), causes oxidative stress, and damages sperm cells (49).

### 2.3 Impact on sperm quality

The high-altitude environment has multiple adverse effects on sperm quality, encompassing key indicators such as sperm density, motility, deformity rate, and genetic material integrity (15, 50).

Previous research shows sperm density in high-altitude areas is lower. Gasco et al. studied male rats at 4340-meter altitude, finding epididymal sperm count decreased from the 7th day (51). Verratti et al. studied 5 Italian men before and after a 19-day high-altitude trek in the Himalayas, showing sperm concentration decreased (50). Their study has clinical value but needs a larger sample size.

Sperm motility, crucial for fertilization, significantly drops in high-altitude environments, reducing the chance of conception (15). The sperm deformity rate also increases, with head and tail deformities affecting sperm function. Luo et al. found altitude impacts sperm genetic material. Sperm mtDNA copy number and nDNA integrity vary with altitude and residence time (52).

Verratti et al. also noted that high-altitude trekking causes oxidative stress imbalance in semen. This imbalance leads to an increase in ROS production. ROS, with its strong oxidative activity, attacks the unsaturated fatty acids on the sperm cell membrane, triggering lipid peroxidation reactions (53). As a result, not only is the integrity and fluidity of the sperm membrane damaged, impairing sperm membrane function and thus affecting sperm motility and fertilization ability, but additionally, the membrane’s barrier function is weakened. This makes it easier for harmful substances to enter the sperm. Furthermore, ROS directly attacks the DNA in the nucleus, causing damage such as DNA strand breaks and base modifications. This affects the integrity of sperm genetic material and severely damages sperm quality (50).

**Figure 1:**
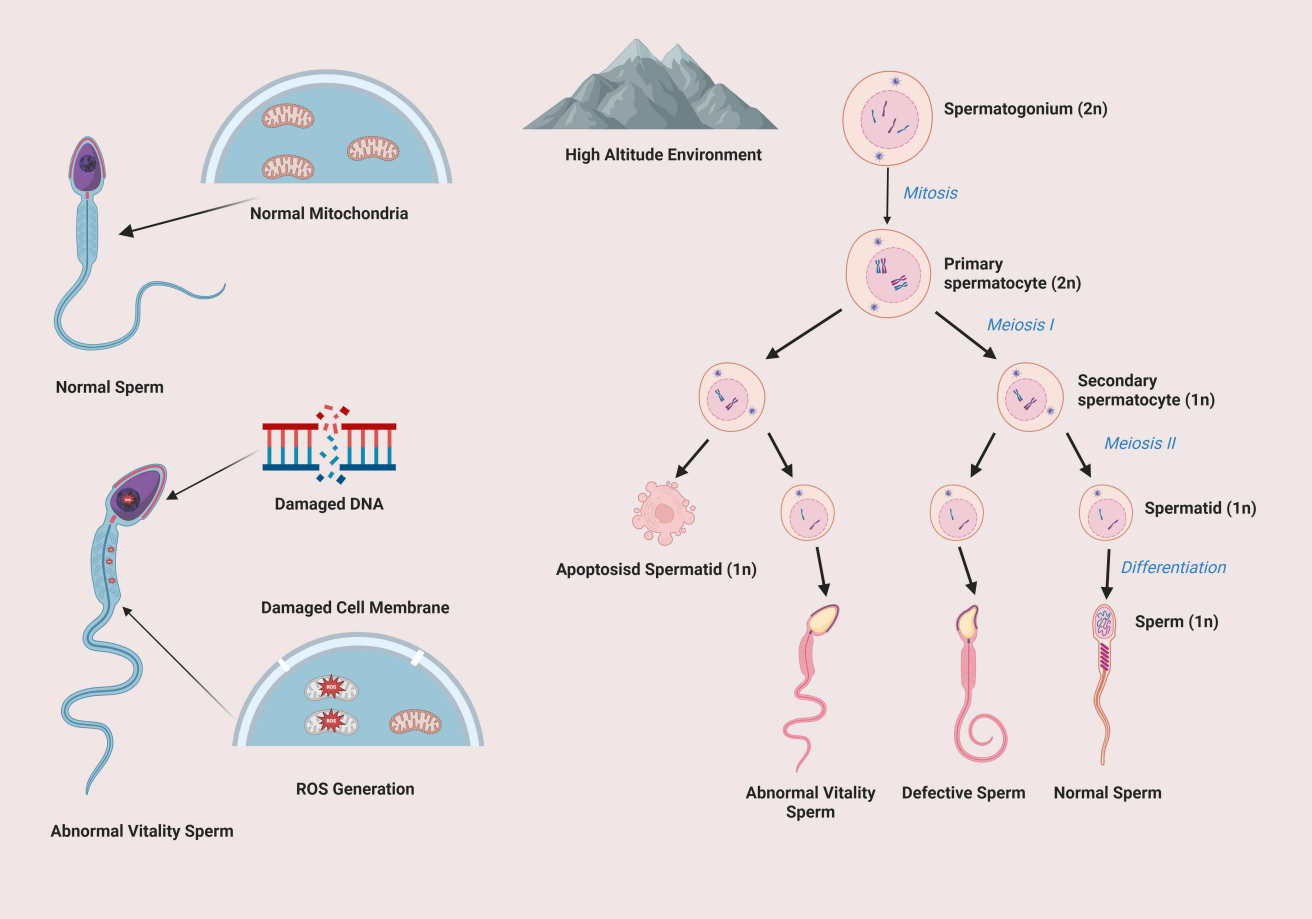
Impact of high altitude on spermatogenic cell development and sperm integrity.

**Figure 1** shows the impact of high-altitude environment on spermatogenesis and sperm quality. In high-altitude conditions, normal spermatogenesis from spermatogonium to sperm is disrupted. Mitochondria are damaged, affecting energy supply. Sperm DNA and cell membranes are also damaged, leading to abnormal sperm vitality and a large number of defective sperm, which seriously impairs sperm quality and male reproductive function.

### 2.4 Impact on sexual function

Sexual dysfunction problems are relatively prevalent among men residing in high-altitude areas for long periods, primarily manifested as decreased libido and erectile dysfunction (50). This phenomenon is caused by the combined action of multiple factors.

From the perspective of the endocrine and reproductive organs, the fluctuations in reproductive hormone levels and testicular tissue damage play a key role in the occurrence of male sexual dysfunction (54–56). Testosterone, as an important hormone for maintaining male libido and sexual response, the decrease in its level will directly weaken male sexual impulse and sexual ability (57, 58). When men are in a high-altitude environment, due to factors such as hypoxia, the synthesis and secretion of testosterone decrease, resulting in a decline in the level of testosterone in the body, which in turn affects sexual function (35, 59). At the same time, testicular tissue damage caused by the high-altitude environment will disrupt the normal physiological function of the reproductive organs, making it difficult to maintain the erectile function in a normal state (17, 60). The testis is an important part of the male reproductive system, and its normal function is of great significance for maintaining erectile function. Testicular tissue damage may affect multiple aspects such as nerve conduction and vascular function, leading to erectile dysfunction (61–63).

The adaptive changes of the nervous system in the high-altitude environment also have a significant impact on sexual function. Verratti et al. conducted a study on three climbers and found that when the altitude rose above 4450 meters, sleep-related erections (SREs) became abnormal, and there was a lack of erections in the hardness ranges of 80-100% and 60-79%. Moreover, the average hardness percentage and the duration of 80-100% hardness gradually decreased with the increase in altitude (64). The underlying mechanism is that nitric oxide (NO) plays a crucial role in the process of penile erection. Under normal nerve stimulation, nerve endings and endothelial cells release NO, which activates guanylate cyclase and promotes the relaxation of penile smooth muscle, thus achieving erection (65, 66). However, in the high-altitude hypoxic environment, the above process is disrupted (67). Hypoxia significantly inhibits the activity of NO synthase (68), resulting in a significant decrease in NO synthesis, and may also reduce the responsiveness of target cells (smooth muscle) to NO, further hindering the relaxation of trabecular smooth muscle and thus inhibiting erectile function (69, 70). In addition, acute hypoxia enhances sympathetic nerve afferent activation and exacerbates vasoconstriction activity (71). Even after the human body has adapted to the high-altitude environment to a certain extent, the excited state of the sympathetic nerve may still persist (72). This continuous excitement will interfere with the normal nerve regulation function and hinder the conduction of sexual excitement, having an adverse impact on the normal performance of sexual function (64).

The **Table 1** presents a comprehensive summary of the specific effects of high-altitude environments on different aspects of the male reproductive system, along with relevant research cases that support these findings.

**Table 1:** Effects of high-altitude environments on the male reproductive system.

Reproductive System	Specific Effects
Reproductive Hormone Levels	- GnRH secretion is inhibited. - FSH and LH levels decrease. - Testosterone synthesis and secretion are affected, and generally decrease with increasing altitude.
Testicular Tissue Morphology	- The structure of seminiferous tubules is disordered. - The arrangement of spermatogenic epithelial cells is abnormal. - The number of spermatogenic cells at all levels decreases, especially spermatocytes and spermatids. - Testosterone levels decrease.
Sperm Quality	- Sperm density decreases. - Sperm motility declines. - The sperm deformity rate increases. - The integrity of sperm genetic material is damaged, and the mtDNA copy number and nDNA integrity change. - Oxidative stress imbalance occurs in semen, with increased ROS, decreased TAC, and reduced radical - scavenging activity.
Sexual Function	- Libido decreases. - Erectile dysfunction occurs.

## 3 Possible mechanisms of high-altitude environment affecting the male reproductive system

### 3.1 Oxidative stress damage

The hypoxic environment in high-altitude areas is one of the key inducers of oxidative stress (73). When the body is in a hypoxic state, the function of the mitochondrial respiratory chain in cells is severely damaged, and the electron transfer process is abnormal, resulting in the massive production of reactive oxygen species (ROS) (74). Under normal circumstances, the body maintains the balance of ROS through its own antioxidant defense system. However, in the high-altitude hypoxic environment, when the production of ROS exceeds the limit of the body’s antioxidant defense capacity, oxidative stress occurs (75–77).

In the male reproductive system, oxidative stress causes particularly serious damage to testicular tissue and sperm (78, 79). ROS has strong oxidizing properties and can attack the unsaturated fatty acids on the sperm cell membrane, triggering lipid peroxidation reactions (53). The products of lipid peroxidation will damage the integrity and fluidity of the sperm membrane, resulting in the impairment of sperm membrane function and then affecting sperm motility and fertilization ability (80–82). Because the integrity of the sperm membrane is crucial for maintaining the stability of the internal environment of sperm and protecting DNA, once the sperm membrane is damaged, harmful substances from the outside are more likely to enter the sperm and cause damage to DNA, thus affecting sperm quality and function (83–85).

In addition, oxidative stress may also interfere with the DNA damage repair mechanism of spermatogenic cells, causing the accumulation of DNA damage in cells and then increasing the apoptosis of spermatogenic cells (79, 86). The normal proliferation and differentiation of spermatogenic cells are the basis of sperm production, and excessive apoptosis of spermatogenic cells will seriously affect the quantity and quality of sperm produced (87, 88). In a study of male soldiers in high-altitude areas, it was found that when they were exposed to a hypoxic environment at an altitude of 5380 meters for 6 months, their total sperm count, sperm density, motility, and survival rate all decreased significantly, and the semen liquefaction time was significantly prolonged. After 12 months, the sperm motility, survival rate, and liquefaction time further deteriorated (15). This may be due to the increase in spermatogenic cell damage and apoptosis caused by high-altitude hypoxia-induced oxidative stress, which breaks the balance between the normal proliferation and apoptosis of spermatogenic cells, seriously hinders the sperm production process, and ultimately affects various quality indicators of sperm (89).

Oxidative stress may also affect the secretion of reproductive hormones (90, 91). In the high-altitude environment, the serum testosterone level of male mice may increase in the early stage of hypoxia, which may be a compensatory response of the body to promote adaptation to the hypoxic environment. However, with the continuous extension of hypoxia time, ROS gradually accumulates in testicular cells, leading to damage to Leydig cells and then reducing the secretion of testosterone. This indicates that oxidative stress may affect the function of Leydig cells and interfere with the normal secretion process of reproductive hormones, thus further affecting male reproductive function (92).

**Figure 2:**
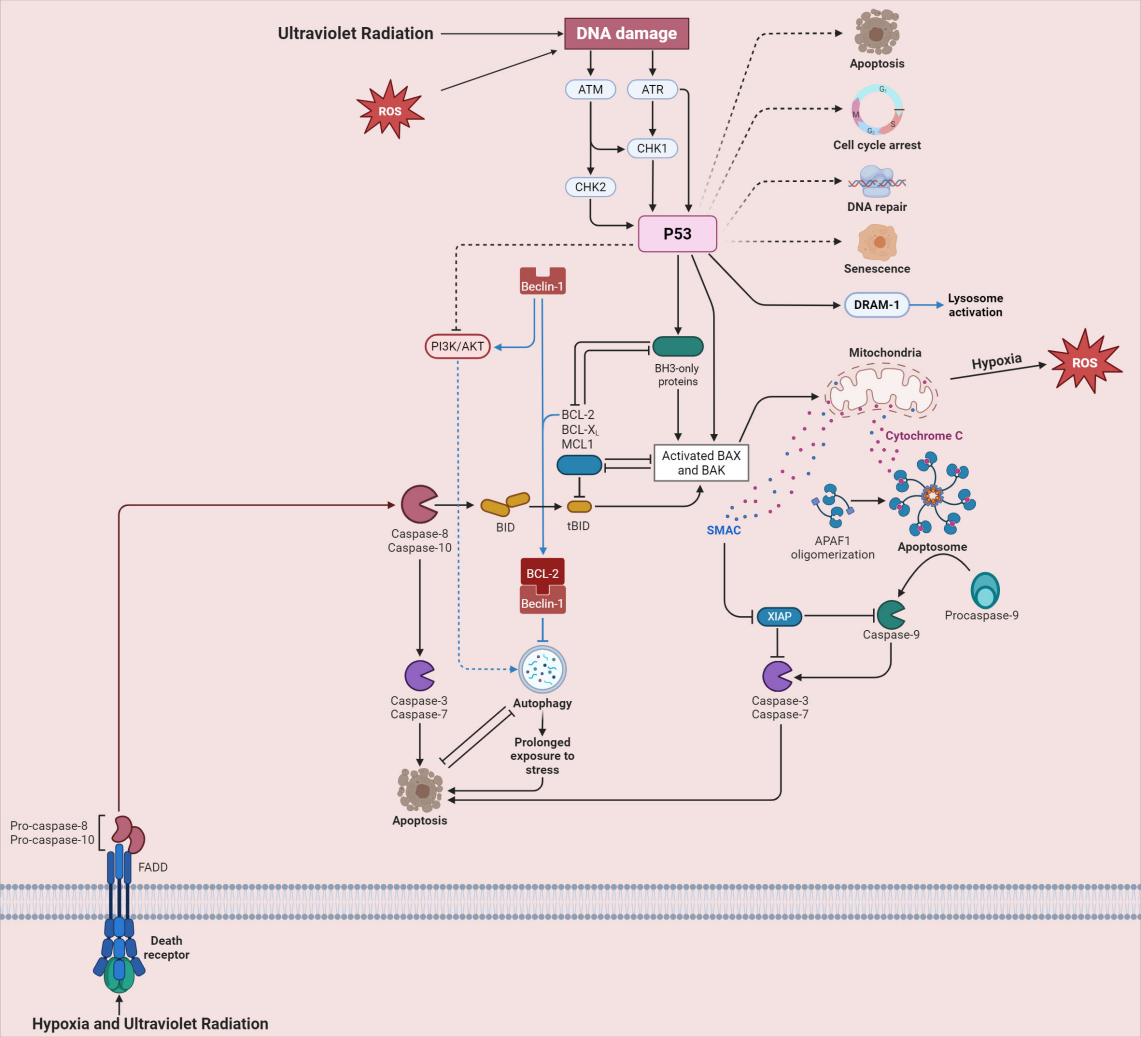
Cellular signaling pathways triggered by ultraviolet radiation and hypoxia in high-altitude areas.

Moreover, as shown in **Figure 2**, hypoxia (93) and ultraviolet radiation (94) can lead to the generation of ROS, which is an important part of the oxidative stress mechanism. Ultraviolet radiation directly inflicts DNA damage (95), activating key signaling molecules ATM and ATR (96). These molecules phosphorylate CHK1 and CHK2 (97), ultimately activating P53 (98). Activated P53 can trigger multiple cellular responses, including apoptosis, cell cycle arrest, and DNA repair (99). In the context of oxidative stress in the male reproductive system, the activation of these pathways may disrupt the normal development and function of spermatogenic cells. Excessive activation of the apoptosis pathway may lead to an increased number of apoptotic spermatogenic cells, reducing sperm production (100). The disruption of the cell cycle arrest and DNA repair pathways may result in the accumulation of damaged DNA in sperm, affecting sperm quality (101).

### 3.2 Apoptosis

The abnormal increase in apoptosis leads to a decrease in the number of spermatogenic cells, seriously disrupting the process of sperm production and development (102). Under normal physiological conditions, the proliferation and apoptosis of spermatogenic cells are in a dynamic balance to maintain the stability of sperm production (103–105). However, in a high-altitude environment, multiple factors lead to an abnormal increase in spermatogenic cell apoptosis, breaking this balance and significantly reducing the number of spermatogenic cells (106, 107). Spermatogenic cells are the basis of sperm production, and the decrease in their number will inevitably affect sperm production and development, resulting in a decrease in sperm quantity and quality and ultimately affecting male reproductive ability (108, 109).

In the testis, apoptosis is an important physiological process, and its functions include maintaining the appropriate ratio of spermatogenic cells to Sertoli cells, clearing abnormal spermatogenic cells, and ensuring quality control of sperm production (110). However, in a high-altitude environment, the abnormal increase in apoptosis caused by factors such as hypoxia greatly disrupts this normal physiological balance (111, 112). Excessive apoptosis of spermatogenic cells leads to a sharp reduction in the number of spermatogenic cells, unable to provide sufficient basal cells for sperm production, resulting in insufficient sperm production. At the same time, a series of biochemical and molecular level changes may occur during apoptosis, which will have a negative impact on the morphology, structure and function of sperm, resulting in a decrease in sperm quality, such as reduced sperm motility and increased deformity rate, and ultimately affecting male reproductive ability (113, 114).

As shown in **Figure 2**, hypoxia and ultraviolet radiation engage with death receptors, spurring the activation of Pro-caspase-8 and Pro-caspase-10 (115). These then activate downstream Caspase-3 and Caspase-7, driving the process of apoptosis (116). The BCL-2 family proteins play a critical role in regulating mitochondrial outer membrane permeabilization (MOMP). When MOMP occurs, cytochrome C is released, forming an azotosome with APAF-1, which activates Caspase-9 and intensifies apoptosis (117). In spermatogenic cells, the imbalance of these apoptotic regulatory factors in the high-altitude environment leads to excessive apoptosis, further exacerbating the damage to male reproductive function (118, 119).

### 3.3 Increase in autophagy

Autophagy is a highly conserved catabolic process in cells (120). Its main function is to wrap damaged proteins, organelles and other substances in the cell through the formation of autophagosomes and transport them to lysosomes for degradation, thereby realizing the recycling of intracellular substances and maintaining the stability of the intracellular environment (121). In the high-altitude environment, the change of autophagy is particularly significant (122, 123).

This increase in autophagy is regulated by multiple signaling pathways (124). The AMP-activated protein kinase (AMPK) pathway is one of the key regulatory pathways (125). In a high-altitude environment, the decrease in oxygen availability leads to a decrease in cellular ATP levels. As a result, AMPK is activated, which in turn promotes the initiation of autophagy (126).

Moreover, the mammalian target of rapamycin (mTOR) signaling pathway also plays a crucial role (127). Under normal conditions, mTOR acts as a negative regulator of autophagy (128). However, in high-altitude environments, the mTOR pathway is inhibited, which releases the suppression of autophagy, thus further promoting the increase of autophagy levels (126, 129, 130).

Autophagy in the high-altitude environment is a double-edged sword. Initially, it serves as a self-protective mechanism for cells. By forming autophagosomes to engulf damaged proteins and organelles and transporting them to lysosomes for degradation, it helps maintain the stability of the intracellular environment, which is beneficial for cells to adapt to the harsh high-altitude environment to a certain extent (131, 132). However, excessive autophagy is detrimental. It can lead to the over-degradation of crucial proteins and organelles in spermatogenic cells (133). Moreover, excessive autophagy may trigger apoptosis in spermatogenic cells, ultimately reducing sperm quality and quantity and impairing male reproductive function. Excessive autophagy may lead to the over-degradation of important proteins and organelles in spermatogenic cells. The over-degradation of mitochondria in spermatogenic cells can disrupt the normal energy metabolism of cells, affecting the process of spermatogenesis (134, 135). This complex behavior of autophagy, with its potential for both protection and harm, is closely related to the regulatory pathways that govern it.

**Figure 2** shows that the PI3K/AKT pathway and autophagy-related protein Beclin-1 are involved in the cellular stress response and interact with apoptosis-related pathways (136, 137). In high-altitude settings, PI3K/AKT activation may regulate autophagy via Beclin-1, influencing its expression and activity (138). This interaction further complicates autophagy regulation in male reproductive cells, and its dysregulation may harm male reproductive function. Further research is needed to comprehensively understand the molecular mechanisms of this interplay and its impact on male reproduction.

## 4 Research status and prospects

At present, research on the damage of the high-altitude environment to the male reproductive system has made certain progress in several aspects. In the field of animal experiments, many studies have observed the impact of the high-altitude environment on the animal reproductive system through simulating the high-altitude environment or directly conducting experiments in high-altitude areas, providing important evidence for revealing relevant mechanisms (13).

However, there are still many deficiencies in existing research. In human studies, large-scale epidemiological investigations on the reproductive health of people in high-altitude areas are scarce. Most studies have small sample sizes, usually involving only a limited number of participants. This makes it hard to cover the wide range of genetic, lifestyle, and environmental differences among high-altitude populations. As a result, the findings may not be applicable to the general high-altitude population. Also, the short observation periods in these studies mean they can’t fully assess the long-term effects on the male reproductive system. Regarding mechanism research, although studies on oxidative stress, apoptosis, and autophagy have been done, many molecular mechanisms remain unclear. The intracellular signaling pathways in high-altitude conditions are complex and interconnected. For instance, the relationship between autophagy and the endoplasmic reticulum stress response in spermatogenic cells under high-altitude stress is not well-understood. When cells are exposed to high-altitude stressors, the endoplasmic reticulum may experience stress, which could potentially trigger autophagy as a compensatory mechanism. However, it’s unclear how the autophagy process is precisely regulated during this endoplasmic reticulum stress response and what the long-term consequences are for spermatogenesis. Additionally, individual differences in adaptability to high-altitude environments are often overlooked. Some people may be more resilient due to genetic factors or healthy lifestyles, but research rarely takes these into account. Research models, both animal and cell models, also have limitations. Animal models can’t completely replicate the human body’s response, and cell models lack the complex environment of the whole organism, making it difficult to accurately reflect the real-world situation.

Future research on the male reproductive system in high-altitude environments should be multi-faceted. It should involve large-scale, multi-center longitudinal epidemiological studies across diverse high-altitude regions, using a unified questionnaire and comprehensive detection indicators that cover reproductive hormones, sperm quality, sexual function, and lifestyle/environmental factors. Long-term follow-up of participants will help observe long-term effects on the male reproductive system, identify damage-related factors, and provide reliable data. Meanwhile, modern molecular biology techniques like gene chips, proteomics, and single-cell sequencing should be used to explore the molecular mechanisms of oxidative stress, apoptosis, and autophagy in the male reproductive system. By analyzing the gene and protein expression profiles of relevant cells, key molecular targets can be uncovered, which is essential for understanding the causes of male reproductive damage and developing new therapies. Finally, based on the results of epidemiological and molecular mechanism research, practical protective and treatment methods should be developed. This includes exploring antioxidant-based interventions for oxidative stress damage and designing regulatory drugs or inhibitors for autophagy and apoptosis. These interventions should first be tested in pre-clinical models and then translated into clinical applications to safeguard the male reproductive system in high-altitude areas and enhance men’s reproductive health.

## 5 Conclusions

In summary, the special environmental factors in high-altitude areas, such as hypoxia, low temperature, and strong ultraviolet radiation, cause extensive and serious damage to the male reproductive system through multiple pathways such as oxidative stress damage, apoptosis, and autophagy. From the disorder of reproductive hormone levels, the change of testicular tissue morphology, to the decline of sperm quality and the appearance of sexual dysfunction, these damages seriously threaten the reproductive health of men in high-altitude areas. Although certain achievements have been made in this field of research, there are still many problems to be solved. In the future, more in-depth and systematic research is needed, strengthen epidemiological investigations of the population, reveal molecular mechanisms, and develop protective and treatment methods to effectively ensure the reproductive health of men in high-altitude areas, promote the sustainable development of high-altitude areas, and provide strong support for human survival and reproduction in high-altitude environments.
